# Enterovirus Genotype EV-104 in Humans, Italy, 2008–2009

**DOI:** 10.3201/eid1606.091533

**Published:** 2010-06

**Authors:** Antonio Piralla, Francesca Rovida, Fausto Baldanti, Giuseppe Gerna

**Affiliations:** Fondazione Istituto Di Ricovero e Cura a Carattere Scientifico Policlinico, San Matteo, Pavia, Italy

**Keywords:** viruses, enterovirus, respiratory infection, phylogenetic analysis, dispatch

## Abstract

In an epidemiologic investigation of respiratory infections in Italy, October 2008–September 2009, we tested samples from patients for respiratory viruses. Human enterovirus genotype EV-104 (identified in Switzerland) was found in 3 immunocompromised and 2 immunocompetent patients. EV-104 is closely related to human rhinoviruses; thus, both types of viruses should be sought in respiratory syndromes.

Human rhinoviruses (HRVs) and enteroviruses (HEVs) have been grouped within the same genus (*Enterovirus*) because of their identical genomic organization and high sequence homology (*1*). However, HRVs infect primarily the respiratory tract, whereas HEVs infect primarily the gastrointestinal tract, from which they can spread to distant sites, such as the central nervous system or myocardial tissue. In addition, HRVs and HEVs differ in several in vitro properties, such as cell tropism, optimal growth temperature, and low pH sensitivity. Notwithstanding these different characteristics, some HEVs possess a respiratory tract tropism similar to that of HRVs; they infect both infants and adults and cause infections of the upper and lower respiratory tracts. Several HEV genotypes, including enterovirus 68; coxsackieviruses (CVs) A9, A21, B2, and B4; and echoviruses 9 and 11, have reportedly been recovered from respiratory secretions or from tissues of patients with bronchitis, bronchiolitis, or pneumonia (*2*–*6*). In an extended epidemiologic study of HEV respiratory infections in children in France in 2008, respiratory syndromes were the second most common HEV-induced pathologic condition after meningitis (31% vs. 44%); HEV caused infections of the lower respiratory tract in 43 (54%) of 79 respiratory infections (*7*).

In Switzerland during 2004–2007, a new enterovirus genotype, EV-104, was reported in association with respiratory signs and symptoms and acute otitis media in 8 children from various regions of the country (*8*). In the past, this virus probably escaped detection because cell cultures lacked sensitivity or because appropriate molecular methods were not used. In addition, only a small percentage of detected HEVs are actually typed. EV-104 belongs to the HEV-C species, and its closest serotypes are CV-A19, CV-A22, and CV-A1 (*9*).

## The Study

During an epidemiologic survey conducted from October 1, 2008, through September 30, 2009, of viral infections of the respiratory tract, in which we collected respiratory samples from all patients admitted to our University Hospital, Fondazione Istituto Di Ricovero e Cura a Carattere Scientifico (IRCCS) Policlinico San Matteo, we detected 5 strains of the new EV-104 genotype. The 5 Pavia (Pav) strains were neither temporally nor epidemiologically related to each other and were recovered from 2 immunocompetent patients (Pav-2 and Pav-4) and 3 immunocompromised patients (Pav-1, Pav-3, and Pav-5) (Table).

**Table:** Characteristics of 5 patients with HEV genotype EV-104 infection, Pavia, Italy, 2009*

Patient no.	Sample no.	Age, y/sex	Hospital department, date of admission	Respiratory secretion Ct value		Clinical symptoms	Underlying disease	Virus identified/ coinfecting virus (Ct)
				HEV	HRV			
Pav-1	NPA/9210	60/M	Hematology outpatient, Apr	29.67	Undetected	Fever, cough, rhinorrhea	HSCT (AML)	EV-104
Pav-2	NPA/9570	62/M	Infectious Diseases outpatient, Apr	23.06	23.05	Chronic rhinopharyngitis	None	EV-104
Pav-3	NPA/11228	7/F	Pediatric Oncohematology, May	33.64	Undetected	Fever, rhinorrhea, conjunctivitis	AML (chemotherapy)	EV-104
Pav-4	NPA/11230	37/F	Infectious Diseases outpatient, May	32.60	Undetected	Chronic rhinopharyngitis	None	EV-104
Pav-5	NPA/13174	2/M	Pediatric Oncohematology, Jun	23.78	31.61	Cough, rhinorrhea, diarrhea, wheezing	HSCT (AML)	EV-104/RSV (18.53)

*HEV, human enterovirus; Ct, cycle threshold; HRV, human rhinovirus; NPA, nasopharyngeal aspirate; HSCT, hematopoietic stem cell transplant; AML, acute myelocytic leukemia; RSV, respiratory syncytial virus.

All respiratory secretion samples (1,500) were routinely tested for respiratory viruses (*10*). In addition, they were tested by real-time reverse transcription–PCR (RT-PCR) for amplification and quantification of both HEVs (*11*) and HRVs (*12*). Primers and probes used for HRV and HEV detection in this study were the following: HRV-forward 5′-CPXGCCZGCGTGGC-3′, HRV-reverse 5′-GAAACACGGACACCCAAAGTA-3′, HRV-probe 5′-TCCTCCGGCCCCTGAATGYGGC-3′, HEV-forward 5′-CCTCCGGCCCCTGA-3′, HEV-reverse 5′-GATTGTCACCATAAGCAGCC-3′, and HEV-probe 5′-CGGAACCGACTACTTTGGGT-3′. However, the HEV assay (*11*) does exhibit several mismatches with EV-104 target.

Using these 2 assays, we detected 5 strains from 5 patients; these strains were amplified to a comparable degree. When these 5 strains were tentatively amplified in the viral protein (VP) 1 region (*13*), no amplicon was obtained, as reported by others (*8*). Amplification products were obtained when typing was attempted by amplifying the VP4/VP2 region (*14*), which yielded a sequence resembling HEV-C genotypes but did not match any of the sequences published in GenBank. Upon request, the new EV-104 strain (*9*) sequence was received from the researchers in Switzerland (GenBank accession no. EU840733). Comparison of the sequences from the strains from Switzerland and the Pav strains showed that the 5 new Pav strains belonged to genotype EV-104 within the HEV-C species (GenBank accession nos. Pav-5, GU722097; Pav-4, GU722098; Pav-3, GU722099; Pav-2, GU722100; Pav-1, GU722101).

**Figure 1:**
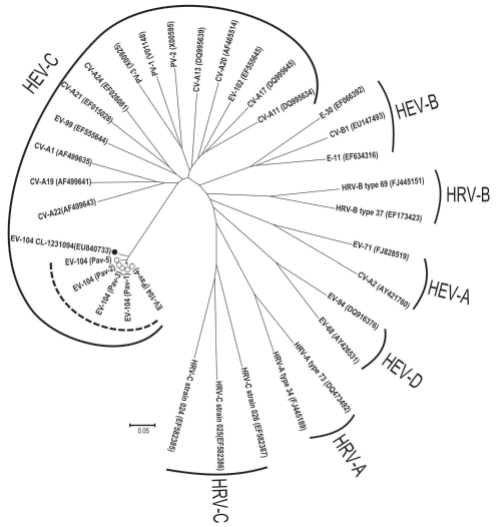
Phylogenetic analysis of the viral protein (VP) 4/VP2 region of the 5 enterovirus 104 (EV-104) strains belonging to the human enterovirus C (HEV-C) species (delimited by circular dotted line), along with the reference strain from Switzerland (GenBank accession no. EU840733). Prototype strains are also reported for the different HEV and human rhinovirus (HRV) species. CV, coxsackievirus; E, echovirus; PV, poliovirus. Scale bar indicates nucleotide substitutions per position.

From the 5 new Pav strains, a 637-nt fragment was obtained. In detail, 181 nt were within the 5′ noncoding region, 207 in VP4, and 249 in VP2. The nucleotide identity within the 5 Pav strains was in the range of 97.9%–99.3%, and the identity with the reference strain from Switzerland was 95.4%–97.0%. Amino acid identity among the Pav strains was 100% for 4 strains; Pav-5 strain showed a 2-aa difference, and the amino acid identity for all Pav strains with the strain found in Switzerland was 98.1%–99.4%. Within the HEV-C species, the closest genotypes are CV-A19, CV-A1, and CV-A22 with a nucleotide identity of 63%, 59%, and 62% and an amino acid identity of 81%, 80%, and 81% with the Pav strains, respectively (Figure 1).

**Figure 2:**
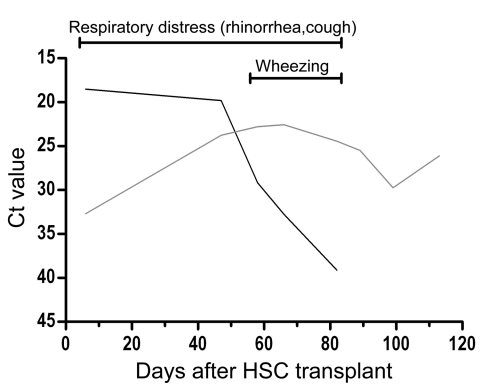
Virologic and clinical follow-up of immunocompromised patient infected with Pavia strain (Pav-5) showing the kinetics of enterovirus 104 (EV-104) and respiratory syncytial virus (RSV) viral loads, along with respiratory symptoms. Starting on day 90 after transplantation, the patient’s clinical symptoms began to disappear in the presence of a substantially unchanged EV-104 viral load in respiratory secretions. Ct, cycle threshold; HSC, hematopoietic stem cells.

In the 2 immunocompetent persons, EV-104 was associated with episodes of chronic rhinopharyngitis, whereas 3 immunocompromised patients exhibited symptoms of acute respiratory tract infection (Table). However, in the patient infected with Pav-5, a hematopoietic stem cell transplant recipient, at the beginning of a 4-month follow-up after the transplant, respiratory syncytial virus was detected in association with EV-104 and was likely responsible for the acute respiratory symptoms (Figure 2) in the immediate posttransplant period. Subsequently, respiratory syncytial virus disappeared, while EV-104 remained at the same stable level (cycle threshold 24–29) during the entire follow-up period. In the last month of the follow-up period, EV-104 was not associated with any clinical or respiratory symptoms. Because we did not grow the virus in cell cultures, we cannot exclude that our real-time RT-PCR assay may have detected only RNA left over from the EV-104 infection. Even in the patient infected with Pav-5, we could not detect any mutation in several samples examined during the 4 months of follow-up.

## Conclusions

The 5 EV-104 strains from Italy, along with the reference strain from Switzerland, form a separate clade within the HEV-C species, which includes 3 polioviruses, several CV-A genotypes, EV-99, and EV-102 (Figure 1). The new HEV genotype confirms the close relationship between HRVs and HEVs, and these findings suggest that both species of viruses should be sought in respiratory syndromes (*8*). Although the EV-104 strains detected in Switzerland were in children from different regions and covered a period of 4 years (*8*), our 5 strains were detected during a short period of the same year and in the same geographic area; the patients, however, had presumably not been in contact with one another. However, population-based studies are needed to infer the actual prevalence of EV-104 infections.

In the patients reported here, only the upper respiratory tract was involved in the EV-104 infection, whereas in the study in Switzerland, otitis media and pneumonia were also reported. Obviously, the range of EV-104 pathogenicity will have to be defined in an extended clinical and epidemiologic survey. However, virus detection in patient Pav-5 in the absence of clinical symptoms indicates a potential nonpathogenic role for this virus, as is already known for HEVs, HRVs, and other respiratory viruses. In contrast, the sustained persistence of EV-104 in the respiratory tract of patient Pav-5 indicates that, at least in immunocompromised patients, virus can be shed for a long period, as has been shown for other respiratory viruses in this patient population (*15*). We found also that the 2 immunocompetent persons (Pav-2 and Pav-4) had a chronic respiratory infection; however, because of limited sampling, the persistence of EV-104 was not shown.

From a methodologic standpoint, we believe it is reasonable to conclude that, although only VP1 has been fully validated in multiple laboratories as the optimal typing region, other genome regions that code for capsid proteins may be amplified to enable typing of known or even unknown HEV genotypes. This method could facilitate the detection of new virus genotypes.
